# Single Carrier with Frequency Domain Equalization for Synthetic Aperture Underwater Acoustic Communications

**DOI:** 10.3390/s17071584

**Published:** 2017-07-06

**Authors:** Chengbing He, Rui Xi, Han Wang, Lianyou Jing, Wentao Shi, Qunfei Zhang

**Affiliations:** School of Marine Science and Technology, Northwestern Polytechnical University, Xi’an 710072, China; xirui@mail.nwpu.edu.cn (R.X.); whan@mail.nwpu.edu.cn (H.W.); jingly369@mail.nwpu.edu.cn (L.J.); swt@nwpu.edu.cn (W.S.); zhangqf@nwpu.edu.cn (Q.Z.)

**Keywords:** underwater acoustic communications, SC-FDE, channel equalization, synthetic aperture

## Abstract

Phase-coherent underwater acoustic (UWA) communication systems typically employ multiple hydrophones in the receiver to achieve spatial diversity gain. However, small underwater platforms can only carry a single transducer which can not provide spatial diversity gain. In this paper, we propose single-carrier with frequency domain equalization (SC-FDE) for phase-coherent synthetic aperture acoustic communications in which a virtual array is generated by the relative motion between the transmitter and the receiver. This paper presents synthetic aperture acoustic communication results using SC-FDE through data collected during a lake experiment in January 2016. The performance of two receiver algorithms is analyzed and compared, including the frequency domain equalizer (FDE) and the hybrid time frequency domain equalizer (HTFDE). The distances between the transmitter and the receiver in the experiment were about 5 km. The bit error rate (BER) and output signal-to-noise ratio (SNR) performances with different receiver elements and transmission numbers were presented. After combining multiple transmissions, error-free reception using a convolution code with a data rate of 8 kbps was demonstrated.

## 1. Introduction

Reliable underwater acoustic communications are challenging because of the limited bandwidths, time-varying multipath delay, double-selective channel fading, and strong background noise [1]. For example, the intersymbol interference (ISI) could span tens or even hundreds of symbol durations. Many approaches have been proposed for underwater acoustic (UWA) communications over the last two decades, including the single carrier with time-domain decision feedback equalizer (SC-TDE) [2,3], orthogonal frequency-division multiplexing (OFDM) [4], single-carrier with frequency domain equalization (SC-FDE) [5,6,7,8], and time reversal communications [9,10].

SC-FDE is a promising low-complexity approach for mitigating intersymbol interference (ISI). The main advantage of SC-FDE is its ability to cope with long delay spread channels with single-tap equalizers. In addition, the estimated channels can be updated easily via pilot signals on a block-by-blocks. Compared to time-domain equalization, SC-FDE has lower computational complexity [11]. Compared to OFDM, SC-FDE exhibits similar or better performance with similar computational complexity while avoiding OFDM’s high peak-to-average power ratio (PAPR) and sensitivity to carrier frequency offset (CFO) [11]. Therefore, SC-FDE has been proposed for IEEE 802.16e [11,12,13] and high-rate underwater acoustic communications [5,6,7,8]. In our previous work, a hybrid time frequency domain equalizer (HT-FDE) is proposed to combat the residual ISI in single carrier UWA communication systems [7].

In phase-coherent UWA communication, multichannel receivers are usually employed to enhance signal-to-noise ratios, mitigate channel fading effects, and reduce the ISI. Spatial diversity assumes the received multichannel signals are independent and the probability that all of them fade at the same time is small. To obtain spatial diversity gain, the optimal element spacing for the receiving array is required on the order of three to four wavelengths for vertical arrays and on the order of 30 to 60 wavelengths for horizontal arrays [14]. Recently, phase-coherent UWA communication has been considered for some practical applications, such as underwater unmanned vehicles (UUV) and gliders. The aforementioned optimal element space requirements may be unsuitable for these small underwater platforms.

Several approaches to achieve phase coherent UWA communication with a single receiver have been developed in recent years [15,16,17,18,19,20,21]. In synthetic aperture communications, only one or a few hydrophones are employed on the underwater platforms. A virtual array is generated by the relative motion between the transmitter and the receiver to achieve spatial diversity gain with reduced size and cost. Non-coherent synthetic aperture time-reversal communications in shallow water was demonstrated at sea where only one transmitter and one receiver were required [15]. The benefit of this system is that it requires only two transducers, significantly decreasing the technical cost compared to typical time reversal communication schemes. The use of a single receiver that combined three consecutive receptions indicated that the synthetic aperture communication is feasible at 9200 km range [16]. High-rate coherent synthetic aperture communications in shallow water at two different bandwidths was presented in [17]. The authors combined two to five consecutive transmissions successfully. They provided an effective data rate of 30/M kbits/s (M is the transmission number) with 8-phase shift keying (PSK) modulations at about a 3.5 km range in shallow water. A glider with a single hydrophone is used for long-range acoustic communication in deep water [18]. This system improved the performance even when individual receptions had low signal-to-noise ratios (SNR). The authors used a binary phase shift keying (BPSK) modulation and time reversal with decision feedback equalization (TR-DFE) with a matched pursuit sparse channel estimation algorithm. The communication range was up to 200 km in deep water. Low-density parity-check (LDPC) coded OFDM synthetic aperture communication was demonstrated in shallow water [19]. Combining multiple transmissions achieves an error-free reception, confirming the feasibility of coherent synthetic communications using OFDM. The source was towed at a speed of three knots at ranges between 600 m and 6 km while a vertical received array was moored in water 106 m deep [19]. A source diversity was considered for phase-coherent UWA communications for UUV with a single receiver element using distributed nodes of underwater acoustic networks [14]. An experiment on passive synthetic aperture time reversal communications was demonstrated via a shallow sea trial [20]. Motion compensation using all-phase fast Fourier transform (FFT) was presented for synthetic aperture spread spectrum underwater acoustic communication [21].

The contributions of this paper include the following: (1) we proposed a SC-FDE approach for synthetic aperture UWA communications; (2) we analyzed the performances of two receiver algorithms, FDE and hybrid time frequency domain equalizer (HTFDE) using data collected from a lake experiment; and (3) we analyzed and compared the influence of receiver element number and transmission number on the performance of the SC-FDE based synthetic aperture communication method.

The rest of this paper is organized as follows. The system model is introduced in Section 1. Section 2 reviews two receiver algorithms of SC-FDE, including FDE and HTFDE. We then present experiment results in Section 3. Finally, we conclude the study in Section 4.

## 2. System Model

### 2.1. SC-FDE Signal

Consider a pseudorandom noise-based (PN) single-carrier block transmission in which the modulated complex-value data streams are divided into blocks of size N−P. We denote data block as ${\left\{x_{m}\right\}}_{m=0}^{N-P-1}$. Each block is extended with a known PN sequence of length *P* to obtain blocks of size *N*, which is expressed as

(1) $$s={\left[x_{0},x_{1},\ldots ,x_{N-P-1},c_{0},c_{1},\ldots ,c_{P-1}\right]}^{T},$$

where ${\left\{c_{n}\right\}}_{n=0}^{P-1}$ is the PN sequence of length *P* and ${\left(\cdot \right)}^{T}$ represents matrix transpose. The same known PN sequence is inserted at the beginning of the transmission frame.

**Figure 1:**
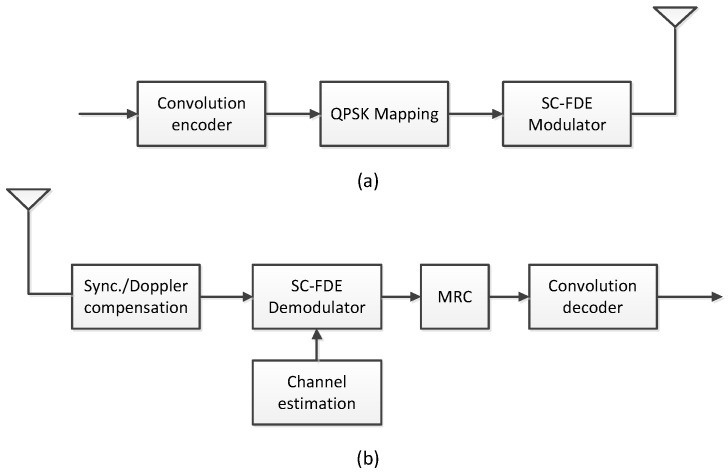
Block diagram: (**a**) transmitter and (**b**) receiver.

The known PN sequence in the previous block can be viewed as the cyclic prefix (CP) of the current transmission block. Assuming that the maximum multipath delay spread is shorter than the PN symbol’s duration, the received signal $r={\left[r_{0},r_{1},...r_{N-1}\right]}^{T}$ is treated as the circular convolution between the transmission data block and the channel impulse response (CIR). Consequently, this transmission format implements a single carrier communication that allows us to exploit the benefits of the frequency domain equalization at the receiver without having the high PAPR of OFDM. A block SC-FDE diagram for the transmitter is shown in Figure 1a. First, an information bit sequence is encoded by a 1/2 rate convolution code (the industry standard convolution code (171, 133)). We then mapped the sequence into symbols using quadrature phase shift keying (QPSK). The SC-FDE system specifications are listed in Table 1. Each SC-FDE block contains 386 information bits or 772 convolution coded bits.

### 2.2. Channel

Assume that the channel is invariant over each block duration. The CIR at block k between the transmitter and the receiver transducer in the discrete time domain is modeled as

(2) $$h\left(k\right)=\sum_{l=0}^{L-1}h_{l}\delta \left(k-l\right),$$

where *k* denotes the *k*-th block and *L* is the channel order. On the receiver side, the Doppler is usually compensated by resampling of the incoming signal [22,23]. Let $r_{k}\left(n\right)$ be the received baseband signal at the *k*-th hydrophone after resampling processing as follows:

(3) $$r_{k}\left(n\right)=e^{j(2\pi f_{k}nT_{s}+\phi_{k,0})}\sum_{l=0}^{L-1}h\left(k,l\right)x_{k}\left(n-l\right)+w\left(k,n\right),$$

where $T_{s}$ is the data symbol duration; $f_{k}$ is the Doppler shift in *k*-th block, caused by relative motion between transceivers, A/D, D/A sampling, variant water current, etc.; $\phi_{k,0}$ is phase rotation caused by the symbol timing offset in the *k*-th block; and w(k,n) is additional white Gaussian noise with power $\sigma^{2}$. The *k*-th received block is expressed as

(4) $$r\left(k\right)={\left[r\left(k,0\right),r\left(k,1\right),\ldots ,r\left(k,N-1\right)\right]}^{T}.$$

For simplicity and to avoid a loss of generality, we eliminated the block index *k*. The received data block is

(5) r=Dhx+w,

where $w={\left[w\left(0\right),w\left(1\right),\ldots ,w\left(N-1\right)\right]}^{T}$ is the noise vector, and D is a diagonal rotated-phase matrix caused by the Doppler shift, which is expressed as

(6) $$D=\mathrm{diag}\left\{e^{j\phi_{k,0}},e^{j(2\pi f_{k}2T_{s}+\phi_{k,0})},\ldots ,e^{j(2\pi f_{k}2\left(N-1\right)T_{s}+\phi_{k,0})}\right\},$$

where

(7) $$\begin{array}{c} h=\left[\begin{array}{c} h_{0}\;0\;\;\ldots \;h_{L-1}\;\ldots \;h_{1}\\ h_{1}\;h_{0}\;\ldots \;\ldots \;\;\;\ldots \;h_{2}\\ \;\vdots \;\ddots \;\ddots \;\ddots \;\;\;\ddots \;\;\vdots \\ h_{L-1}\ldots \;h_{1}\;\;h_{0}\;\;\;0\;\;\;0\\ 0\;\;\ldots \;\ldots \;\ldots \;\;\;\ldots \;0\\ \;\vdots \;\;\ddots \;\ddots \;\;\ddots \;\;\ddots \;\vdots \\ 0\;\ldots \;h_{L-1}\;\ldots \;h_{1}\;\;h_{0} \end{array}\right] \end{array}$$

is an N×N circulant matrix.

### 2.3. Receiver

**Figure 2:**
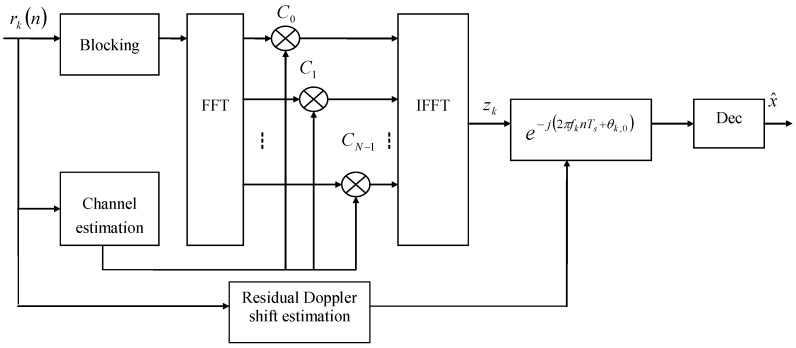
Basic FDE receiver structure for the single carrier-system.

A block diagram for the receiver is shown in Figure 1b. The SC-FDE demodulator in Figure 2 consists of a down-converter, PN remover, FFT, channel estimator, frequency domain equalizer, and inverse Fast Fourier Transform (IFFT). In the receiver, the data is converted to the frequency-domain from time-domain via FFT operation, and then frequency-domain equalization is conducted. The equalized signal is converted back to a time-domain signal. The frequency domain expression of Equation (5) is given as

(8) R=DΛX+W.

In summary, the FDE part consists of a rotated-phase compensation, channel estimation, and frequency domain equalization.

Channel estimation plays a critical role in underwater acoustic communications [24,25,26]. We carried out channel estimation using the known PN symbols block-by-block. The underwater channel often exhibits sparse characteristics, which cause the channel to have a large delay spread but many of the channel taps to have negligible values. We used the improved proportionate normalized least mean squares (IPNLMS) method in the receiver because it has been shown to yield a robust estimation for sparse channels [27,28]. We took this sparseness into account to apply the IPNLMS algorithm to the UWA channel estimation. The IPNLMS algorithm showed not only robust performance in non-sparse channels but also better performance than LMS in sparse channels.

For the *i*-th block, the estimated channel impulse response vector is denoted by ${\hat{h}}_{i}$. In practice, we accounted for the time-varying property of the underwater acoustic channel by averaging the estimated channels from the (i−1)-th and *i*-th blocks:

(9) $${\hat{h}}_{i}=\frac{{\hat{h}}_{i-1}^{{}^{\prime }}+{\hat{h}}_{i}^{{}^{\prime }}}{2}.$$

**Figure 3:**
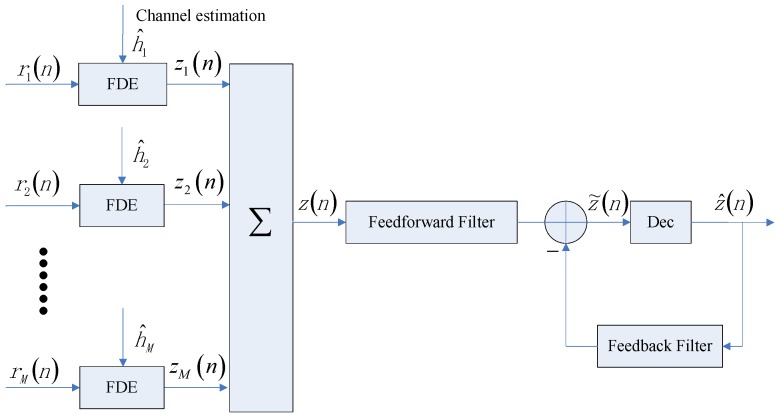
Hybrid time frequency domain equalizer (HTFDE).

We achieved diversity in the combination of different transmissions by maximal ratio combining (MRC) [19,27]. The basic FDE assumes the channel is invariant in one block. For channel variations in one block or channel estimation errors, there is residual ISI in the MRC output of multichannel frequency domain equalizers. We then used a single-channel adaptive time domain DFE to combat the residual ISI, as shown in Figure 3. We use the recursive least square (RLS) algorithm to update the tap coefficients of DFE.

## 3. Communication Performance

### 3.1. DJK16

We start by describing the 2016 Danjiangkou (DJK16) lake experiment, in which we transmitted QPSK signals from one source to eight receivers. We conducted the DJK16 experiment in Danjiangkou Lake, Henan Province, China, in January 2016. The water depth was 45–50 m. The source was deployed at 20 m below the surface. The receiver array had eight elements that were uniformly spaced at 0.25 m apart and was deployed at depths between 18–20 m. The data were transmitted from the source using QPSK modulation with a carrier frequency of 6 kHz and a bandwidth of 4 kHz. The distances between transmitter and receivers ranged from 4.98 to 5.20 km. The longest distance between different transmitter locations was about 720 m, while the shortest was about 20 m.

### 3.2. Packet Design

**Figure 4:**
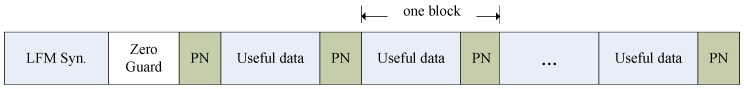
Transmitted signal structure.

Each packet consisted of a 200 ms-long linear frequency modulated (LFM) waveform, 100 ms zero padding period, followed by 32 SC-FDE blocks, each of which was 128 ms long. Each packet lasted 4.428 s and included 16,510 symbols. The total transmitted signal included five different packets, and its duration was about 22.14 s. We transmitted identical signals at each location. The LFM waveform at the beginning of the packet was used for synchronization and Doppler shift estimation. The coded data rate is about 2.79 kbps if we consider the synchronization LFM waveform, the additional zero-padding and the extra PN length before the first block. The structured signal was transmitted as shown in Figure 4.

The detailed parameters of the communication system used in the lake experiment are shown in Table 1. The order of feedforward and feedback filters in HTFDE were both 32, respectively. We used the fractionally spaced equalizer (two samples per symbol) for the feedforward filter. The RLS forgetting factor λ in the DFE was 0.999. We corrected the residual phase offset using a second-order digital phase locked loop (DPLL) embedded in the DFE. Both $K_{1}$ and $K_{2}$ in DPLL were 0.005. The first PN sequence is used as training sequence.

### 3.3. Channels

**Figure 5:**
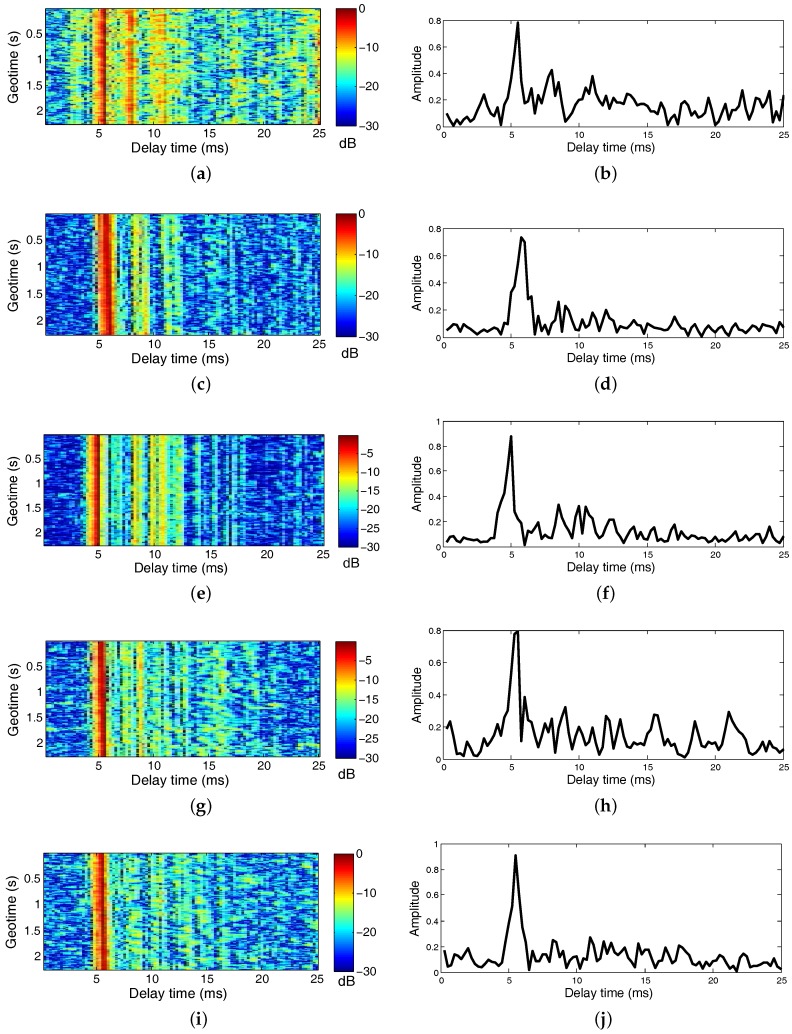
Examples of estimated CIRs for the DJK16 data at five transmissions. The left panel shows CIRs varies as a function of geotime, the right panel show a snapshot of the CIRs. (**a**,**b**) shows the CIRs from 5.2 km; (**c**,**d**) shows the CIRs from 4.98 km; (**e**,**f**) shows the CIRs from 5.09 km; (**g**,**h**) shows the CIRs from 5.18 km; and (**i**,**j**) shows the CIRs from 5.0 km, respectively.

Examples of channel impulse responses (CIRs) are shown as a function of geotime in the left panel of Figure 5 for the transmissions from the source to Rx# 1 during the five transmissions. The CIRs are unique because of the signals’ different times and locations. A snapshot of the CIRs for each transmission at the five locations is shown in the right panel of Figure 5. The approximately 20 ms of delay spread (less than 31.5 ms) in the channels corresponds to intersymbol interference of about 80 symbols at the symbol rate of R = 1/T = 4000 symbols/s. The DJK16 channels varied slowly with time over 2.5 s and exhibits sparse characteristics. We used an IPNLMS algorithm in the analysis because it can yield robust estimates for sparse UWA channels.

**Figure 6:**
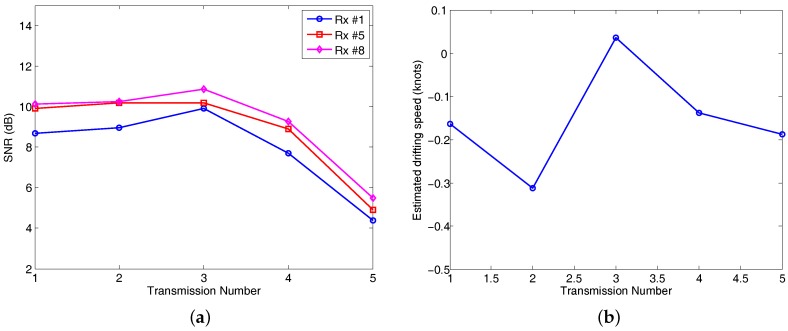
Estimated input SNRs (**a**), and drifting speed (**b**).

The received SNRs of the five transmissions ranged from 4 dB to 11 dB as shown in Figure 6a. The SNR of the fifth transmission was the lowest, about 4 dB. The estimated SNRs of the bottom receiver element (Rx #8) were slightly higher than the SNRs for the top element (Rx #1).

The estimated transmitter drifting speed (Doppler estimation) for the five transmissions is shown in Figure 6b. The drifting speed ranged from −0.32 knots to 0.05 knots, reflecting the Doppler shift from −0.64 Hz to 0.01 Hz at the center frequency of 6000 Hz.

### 3.4. Results

**Figure 7:**
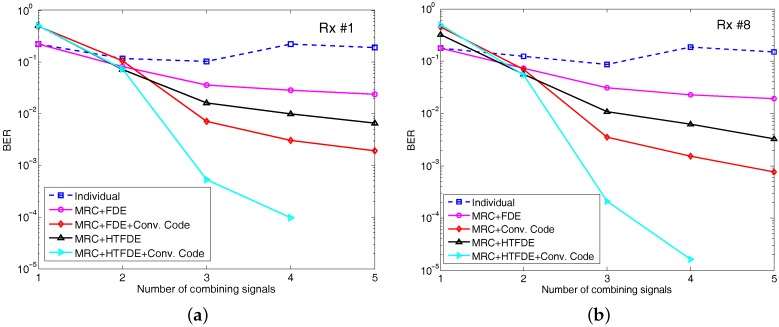
Performance comparison between (**a**) top (Rx #1) and (**b**) bottom (Rx #8). Three different approaches are compared: (1) individual performance without combining; (2) MRC with FDE; (3) MRC with convolution code; (4) MRC with HTFDE; and (5) MRC with HTFDE and convolution code.

We used the uncoded bit error rate (BER) and the output SNR as the performance metrics. We investigated the impact of distributed diversity combining on the performances of BERs of Rx #1, as shown in Figure 7a as a function of the number of transmissions combined. We compared five different approaches: (1) individual performance without combining; (2) MRC with FDE; (3) MRC with convolution code; (4) MRC with HTFDE; and (5) MRC with HTFDE and convolution code.

The BERs without combining were quite high (i.e., 13–22%). From one to three transmissions, the BER performance improved significantly with increasing diversity. After three transmissions (e.g., four and five transmissions), the performance improvement for the uncoded system was slight. For the MRC and FDE method, after five transmission combinations, the uncoded BER of Rx #1 was $2.36\times 10^{-2}$, whereas the coded BER of Rx #1 with the convolution code was $1.9\times 10^{-3}$. For the MRC and HTFDE method, the uncoded BER of Rx #1 was $6.6\times 10^{-3}$, whereas the coded BER with convolution code achieved error-free reception by five transmission combinations. Once the BER of the MRC-FDE was less than a certain level (10%), significant performance enhancement using HTFDE is achieved. To check the consistency of the results, we also plotted the performances of Rx #8 in Figure 7b. Other receivers at different depths achieved similar results.

**Figure 8:**
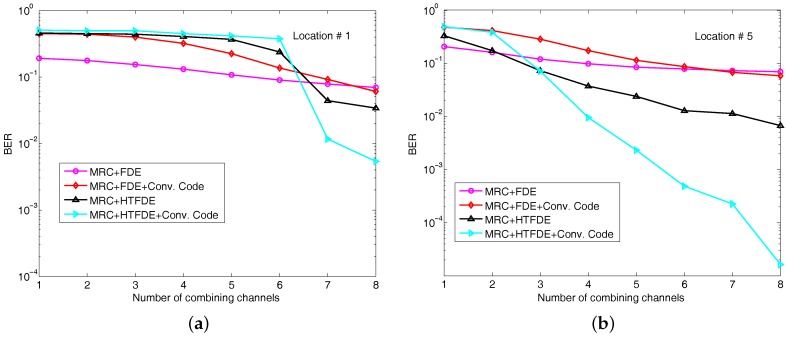
BER performance using conventional multichannel FDE and HTFDE at one location: (**a**) Location #1 and (**b**) Location #5.

For comparison, we also plotted the BER results of conventional multichannel equalization with 1–8 channels for the first and fifth location in Figure 8. With an 8-channel receiver in one location, the uncoded BER was about $7\times 10^{-2}$ using MRC and FDE and the coded BER was $2\times 10^{-2}$. The uncoded BER using MRC and HTFDE is $3.4\times 10^{-2}$ and the coded BER is $5.3\times 10^{-3}$. As shown in Figure 8, the BER improvement using the five receiver elements at one location was far smaller than the improvement using the data of Rx #1 from five transmissions. This occurred because the channel correlation between the different elements was higher than the correlation between different transmissions.

**Figure 9:**
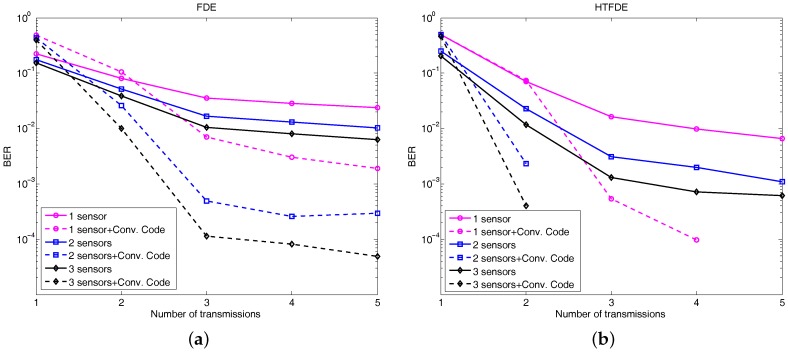
BER performances as a function of the number of transmissions and the number of elements used: (**a**) the performance with MRC-FDE and convolution code and (**b**) the performance with MRC-HTFDE and convolution code.

A linear array consisting of eight elements was used in the lake experiment. Figure 9 shows the BER performance using up to three contiguous elements with five transmissions. When we used elements from 1 to 3, the uncoded BER of MRC-FDE decreased from $1.5\times 10^{-2}$ to $3\times 10^{-3}$, whereas the uncoded BER of MRC-HTFDE decreased from $6.6\times 10^{-3}$ to $6.1\times 10^{-4}$. We demonstrated that error-free using three elements combined three transmissions, especially using MRC-HTFDE.

**Figure 10:**
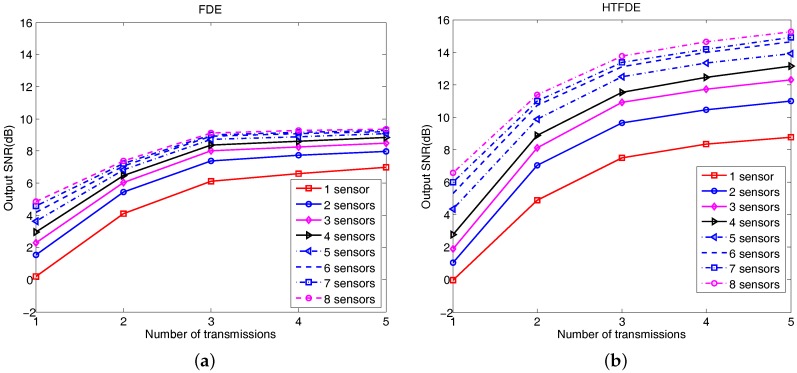
Output SNR as a function of the number of transmissions and the number of elements used. (**a**) FDE results and (**b**) HTFDE results.

Figure 10 shows the output SNRs using different elements and transmission numbers using MRC-FDE and MRC-HTFDE methods. With the same transmission number, the output SNR improvement reduced as we used more receiver elements. The output SNRs of the three transmissions with three receiver elements were 8 dB for MRC-FDE and 10.9 dB for MRC-HTFDE. With three contiguous elements, the 0.5-m-long linear array with three transmissions achieved error-free reception. Such a three-element array is low cost and acceptable for small underwater platforms, like UUVs. Through the use of multiple elements, the BER can be reduced significantly, thus reducing the required number of transmissions, shown in Figure 10.

**Figure 11:**
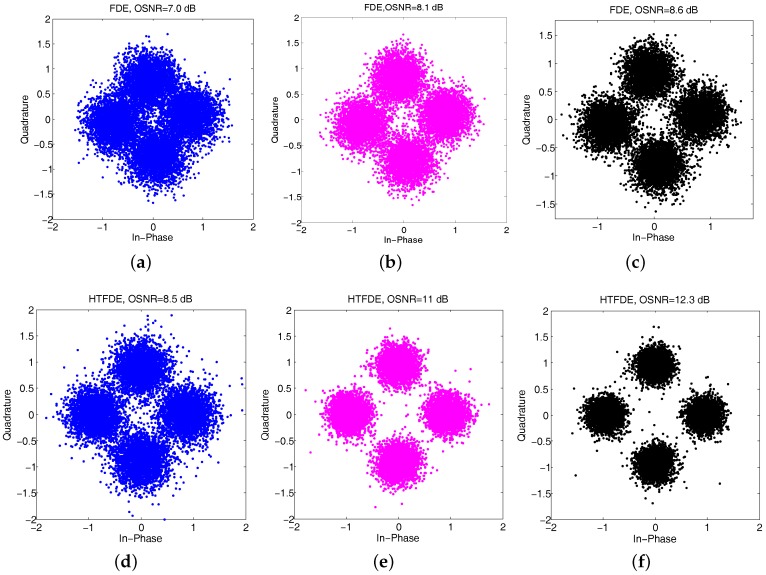
Scatterplot of the equalized first packet signal using the five transmissions with conventional FDE and HTFDE: (**a**) using the 1st element with FDE; (**b**) using elements 1 and 2 with FDE; (**c**) using elements 1 to 3 with FDE; (**d**) using the 1st element with HTFDE; (**e**) using elements 1 and 2 with HTFDE; (**f**) using elements 1 to 3 with HTFDE.

To illustrate the improvements using different receiver elements, we present scatterplots of equalized QPSK signals of the first packet using the IPNLMS channel estimation algorithm in Figure 11, which includes the following: (a) MRC-FDE using the 1st receiver element; (b) MRC-FDE using receiver elements 1 and 2; (c) MRC-FDE using receiver elements 1 to 3; (d) MRC-HTFDE using the 1st receiver element; (e) MRC-HTFDE using receiver elements 1 and 2; and (f) MRC-HTFDE using receiver elements 1 to 3. The top panel displays the results of using MCR-FDE; the bottom panel displays the results of using MRC-HTFDE. The output SNRs for MRC-FDE were 7.0 dB, 8.1 dB, and 8.6 dB, whereas the output SNRs for MRC-HTFDE were 8.5 dB, 11 dB, and 12.3 dB, respectively. The output SNR improvements were 1.5 dB, 3.1 dB, and 3.7 dB.

## 4. Conclusions

In this paper, we propose an SC-FDE scheme for synthetic aperture communications. The transmitter sent a particular set of data at different locations. Spatial and temporal diversities are obtained using multiple transmission combinations. We exploited two receiver algorithms, MRC-FDE and MRC-HTFDE, and we demonstrated error-free receptions after five transmission combinations using convolution decoding. The addition of a few receiver elements increased the output SNR and decreased the BER significantly and caused a slight increase in the cost of the system.

## Figures and Tables

**Table 1:** Single carrier with frequency domain equalization communication system specifications.

NFFT size	512
Number of data symbol	386
PN length	126
Bandwidth	4 kHz
PN duration	31 ms
Symbol duration including PN	128 ms
Uncoded data rate (QPSK)	5580 bps
Uncoded spectral efficiency (QPSK)	1.5 bits/s/Hz
Coded data rate (QPSK)	2790 bps
Coded spectral efficiency (QPSK)	0.70 bits/s/Hz
Convolution code	(171, 133)

## References

[1] Kilfoyle D.B., Baggeroer A.B. (2000). The state of the art in underwater acoustic telemetry. IEEE J. Ocean. Eng..

[2] Stojanovic M., Catipovic J.A., Proakis J.G. (1994). Phase-coherent digital communications for underwater acoustic channels. IEEE J. Ocean. Eng..

[3] Stojanovic M. (2008). Efficient processing of acoustic signals for high-rate information transmission over sparse underwater channels. Phys. Commun..

[4] Li B., Zhou S., Stojanovic M., Freitag L., Willett P. (2008). Multicarrier communication over underwater acoustic channels with nonuniform Doppler shifts. IEEE J. Ocean. Eng..

[5] Zheng Y.R., Xiao C., Yang T.C., Yang W.B. (2010). Frequency-domain channel estimation and equalization for shallow-water acoustic communications. Phys. Commun..

[6] He C., Huang J., Zhang Q., Shen X. Single carrier frequency domain equalizer for underwater wireless Communication. Proceedings of the 2009 WRI International Conference on Communications and Mobile Computing.

[7] He C.B., Huang J.G., Zhang Q.F. Hybrid Time-Frequency Domain Equalization for Single-Carrier Underwater Acoustic Communications. Proceedings of the Conference on Under Water Networks WUWNet ’12.

[8] Xia M., Rouseff D., Ritcey J.A., Zou X. (2014). Underwater Acoustic Communication in a Highly Refractive Environment Using SC-FDE. IEEE J. Ocean. Eng..

[9] Yang T.C. (2005). Correlation-based decision-eeedback equalizer for underwater acoustic communications. IEEE J. Ocean. Eng..

[10] Song A., Badiey M., McDonald V.K., Yang T.C. (2011). Time Reversal Receivers for High Data Rate Acoustic Multiple-Input Multiple-Output Communication. IEEE J. Ocean. Eng..

[11] Falconer D., Ariyavisitakul S.L., Benyamin-Seeyar A., Eidson B. (2002). Frequency domain equalization for single-carrier broadband wireless systems. IEEE Commun. Mag..

[12] (2006). IEEE Standard for Local and Metropolitan Area Networks, Part 16: Air Interface for Fixed and Mobile Broadband Wireless Access Systems.

[13] Pancaldi F., Vitetta G.M., Kalbasi R., Al-Dhahir N., Uysal M., Mheidat H. (2008). Single-carrier frequency domain equalization. IEEE Signal Process. Mag..

[14] Yang T.C., Heaney K.D. Network-Assisted underwater acoustic communications. Proceedings of the Conference on Under Water Networks WUWNet ’12.

[15] Higley W.J., Roux P., Kuperman W.A., Hodgkiss W.S., Song H.C., Akal T., Stevenson M. (2005). Synthetic aperture time-reversal communications in shallow water: Experimental demonstration at sea. J. Acoust. Soc. Am..

[16] Song H.C., Dzieciuch M. (2009). Feasibility of global-scale synthetic aperture communications. J. Acoust. Soc. Am..

[17] Song H.C., Hodgkiss W.S., Kuperman W.A., Akal T., Stevenson M. (2009). High-rate synthetic aperture communications in shallow water. J. Acoust. Soc. Am..

[18] Song H.C., Howe B.M., Brown M.G., Andrew R.K. (2014). Diversity-based acoustic communication with a glider in deep water. J. Acoust. Soc. Am..

[19] Song H.C., Hodgkiss W.S., Kang T. (2011). Multi-carrier synthetic aperture communication in shallow water: Experimental results. J. Acoust. Soc. Am..

[20] Sun B.W., Guo S. (2010). An Experiment On Passive Synthetic Aperture Time Reversal Communications in Shallow Water. AIP Conf. Proc..

[21] Wang Z.J., Yu L., Huang H.N. (2013). Applications of All-phase FFT in Motion Compensation for Synthetic-aperture Underwater Acoustic Communication System. J. Electron. Inf. Technol..

[22] Sharif B.S., Neasham J., Hinton O.R., Adams A.E. (2000). A computationally efficient Doppler compensation system for underwater acoustic communications. IEEE J. Ocean. Eng..

[23] Gendron P.J. (2016). Shallow water acoustic response and platform motion modeling via a hierarchical Gaussian mixture model. J. Acoust. Soc. Am..

[24] Cotter S.F., Rao B.D. (2002). Sparse channel estimation via matching pursuit with application to equalization. IEEE Trans. Commun..

[25] Li W., Preisig C. (2007). Estimation of rapidly time-varying sparse channels. IEEE J. Ocean. Eng..

[26] Berger C.R., Wang Z., Huang J., Zhou S. (2010). Application of compressive sensing to sparse channel estimation. IEEE Commun. Mag..

[27] Meng Q., Huang J., Han J., He C., Ma C. An improved direct adaptive multichannel turbo equalization scheme for underwater communications. Proceedings of the OCEANS 2012—Yeosu.

[28] Pelekanakis K., Chitre M. (2015). Robust Equalization of Mobile Underwater Acoustic Channels. IEEE J. Ocean. Eng..

